# Hydrogenated Amorphous Titania with Engineered Surface Oxygen Vacancy for Efficient Formaldehyde and Dye Removals under Visible-Light Irradiation

**DOI:** 10.3390/nano12050742

**Published:** 2022-02-22

**Authors:** Guang Feng, Mengyun Hu, Botao Wu, Shencheng Shi, Shuai Yuan, Yanan Li, Heping Zeng

**Affiliations:** 1 School of Optical-Electrical and Computer Engineering, University of Shanghai for Science and Technology, Shanghai 200093, China; 191380035@st.usst.edu.cn (G.F.); myhu@phy.ecnu.edu.cn (M.H.); syuan@usst.edu.cn (S.Y.); 2 Chongqing Key Laboratory of Precision Optics, Chongqing Institute of East China Normal University, Chongqing 401120, China; 3 State Key Laboratory of Precision Spectroscopy, East China Normal University, Shanghai 200062, China; btwu@phy.ecnu.edu.cn (B.W.); 51190920037@stu.ecnu.edu.cn (S.S.); 51200920008@stu.ecnu.edu.cn (Y.L.)

**Keywords:** amorphous TiO_2_, oxygen vacancy, bandgap, formaldehyde, visible light

## Abstract

Hydrogenated crystalized TiO_2−x_ with oxygen vacant (O_V_) doping has attracted considerable attraction, owing to its impressive photoactivity. However, amorphous TiO_2_, as a common allotrope of titania, is ignored as a hydrogenated templet. In this work, hydrogenated amorphous TiO_2−x_ (HAm-TiO_2−x_) with engineered surface O_V_ and high surface area (176.7 cm^2^ g^−1^) was first prepared using a unique liquid plasma hydrogenation strategy. In HAm-TiO_2−x_, we found that O_V_ was energetically retained in the subsurface region; in particular, the subsurface O_V_-induced energy level preferred to remain under the conduction band (0.5 eV) to form a conduction band tail and deep trap states, resulting in a narrow bandgap (2.36 eV). With the benefits of abundant light absorption and efficient photocarrier transportation, HAm-TiO_2−x_ coated glass has demonstrated superior visible-light-driven self-cleaning performances. To investigate its formaldehyde photodegradation under harsh indoor conditions, HAm-TiO_2−x_ was used to decompose low-concentration formaldehyde (~0.6 ppm) with weak-visible light (λ = 600 nm, power density = 0.136 mW/cm^2^). Thus, HAm-TiO_2−x_ achieved high quantum efficiency of 3 × 10^−6^ molecules/photon and photoactivity of 92.6%. The adsorption capabilities of O_2_ (−1.42 eV) and HCHO (−1.58 eV) in HAm-TiO_2−x_ are both largely promoted in the presence of subsurface O_V_. The surface reaction pathway and formaldehyde decomposition mechanism over HAm-TiO_2−x_ were finally clarified. This work opened a promising way to fabricate hydrogenated amorphous photocatalysts, which could contribute to visible-light-driven photocatalytic environmental applications.

## 1. Introduction

As a promising and environmentally-friend photocatalyst, titanium dioxide (TiO_2_) has gained widespread traction in the past few decades [1,2,3,4,5]. In order to pursue the best performance in solar energy conversion and utilization, one of the most effective approaches in tuning the band structure and maximizing solar energy capture is O_V_ defect engineering of TiO_2_ [6,7,8,9]. Among considerable strategies in O_V_ defect engineering, the most representative and effective one is thermal hydrogenation of crystallized TiO_2_, which promotes the generation of O_V_-doped disordered surface at crystalline TiO_2_, i.e., a disordered surface at the crystalline core [9,10,11,12]. This unique configuration not only provides additional O_V_-induced states to absorb long-wavelength light, thereby enabling colorful TiO_2−x_, but also encourages photoinduced charge separation to achieve superior visible-light photoactivity [13,14,15]. To obtain photocatalysts with superior efficiency, the polymorph modifications of crystalline titanium dioxide also attracted much attention [16,17]. Alternatively, compared with crystalline TiO_2_, amorphous TiO_2_, which also exhibits an intrinsic narrow bandgap, thermodynamic metastability, and a large surface area, should be a great promising templet for surface hydrogenation [18,19,20]. Nevertheless, inherent drawbacks, including large quantities of bulk defects and inferior solar energy conversion eliminate this possibility. Theoretically, hydrogenated amorphous TiO_2−x_ (HAm-TiO_2−x_) ought to have a narrower bandgap and more surface-O_V_-induced active sites, which are the crucial factors for high-visible photoactivity. However, in practice, thermal hydrogenation using amorphous TiO_2_ is ineluctable to require a long annealing process, which completely transforms the product into hydrogenated crystalline TiO_2−x_ and easily erases the surface O_V_. Given the above challenges in the synthesis theory and technology, hydrogenation of pure amorphous phase TiO_2_ has so far scarcely succeeded. Considering these aspects, the goal of this work is to prepare HAm-TiO_2−x_ with an O_V_-doped disordered surface (disordered surface at the amorphous core) and, particularly, to reveal the fundamental mechanism of the correlation between O_V_ concentration and distribution, electrical structure, optical response, reactive oxygen species (ROS) generation, and its photoactivity, which can discover uncommon electrical and optical properties of HAm-TiO_2−x_ better than traditional hydrogenated crystalline TiO_2−x_.

Formaldehyde, one of the most common indoor air pollutions, is highly poisonous and carcinogenic and is an immense threat to human health [21,22]. Photocatalytic formaldehyde purification is an efficient and safe method that can completely decompose formaldehyde into carbon dioxide and water [23,24,25,26,27]. Current studies on photocatalytic formaldehyde photodegradation are mainly focused on reaction mechanisms, such as peculiar formaldehyde physicochemical adsorption models and complex reaction pathways. In particular, they are focused on the synthesis of advanced photocatalysts with superior formaldehyde degradation performance under standard and ideal experimental conditions, including high power density of UV light source, high concentration formaldehyde, as well as high reaction temperature and pressure. However, in a practical indoor environment the situation is completely inverse: a practical indoor environment provides low-concentration formaldehyde (0.2–0.5 ppm), a weak-visible-light illumination (0.1–0.3 mW/cm^2^), and indoor temperature (25 °C). Photocatalysts with inferior visible-light response and photocarrier transferring cannot actively work under these conditions. More importantly, the ambient durability of photocatalysts should be stressed because of surface fouling/deactivation induced by dust and aerosol particles [28,29]. Herein, a photocatalyst must possess strong visible-light-driven photoactivity, a straightforward reaction pathway, and a self-cleaning surface with long-term stability. With the advantages of narrow bandgap, large specific surface area, and abundant surface O_V_-induced active sites, HAm-TiO_2−x_ could be well suited for realistic formaldehyde purification.

In this work, we first reported the preparation of hydrogenated amorphous TiO_2−x_ nanoparticles and systematically expounded the effects of surface O_V_ on its optical bandgap, surface dynamics of photocarriers, and visible-light photoactivity. After functionalized surface hydrogenation by a liquid plasma strategy, the distribution and concentration of O_V_ in HAm-TiO_2−x_ can be designed well via manipulating the output power of liquid plasma discharge. According to density function theory simulations, one important finding was that the O_V_ concentration determined the existence of deep states in the bandgap, while the accurate energy level for these deep states was decided by the O_V_ distribution. Surface O_V_-induced deep states were formed close to the valence band, whereas subsurface O_V_-induced deep states were mainly generated under the conduction band. With the benefits of narrow bandgap and efficient charge carrier migration, HAm-TiO_2−x_ and its coating exhibited excellent visible-light dye degradation and stability. More importantly, low-concentration formaldehyde (0.6 ppm) was completely decomposed in an ambient environment using HAm-TiO_2−x_. The durability test and apparent quantum efficiency of formaldehyde removal under visible light (λ = 600 nm, power density = 0.136 mW/cm^2^) were explored. In addition, ROS generation, formaldehyde photodegradation pathway, chemical adsorption energy, and photocatalytic mechanism of HAm-TiO_2−x_ were investigated.

## 2. Experimental Section

### 2.1. Reagents and Materials

Titanium foam (99%, 100 mesh) and titanium rods (99%, 3-mm diameter) were acquired from Baoji Hong Xinyuan Metal Material Co., Ltd. (Baoji, China) P25 TiO_2_ was purchased from Degussa (Evonik, Essen, Germany).

### 2.2. Preparation of HAm-TiO_2−x_

**Figure 1:**
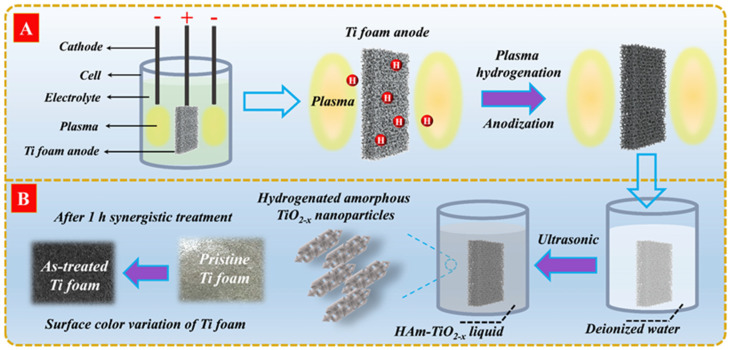
(**A**) Synthesis setup of producing HAm-TiO_2−x_, and (**B**) the generation process of HAm-TiO_2−x_ nanoparticles.

One piece of anodic titanium foam (20 × 20 × 2 mm^3^) and two cathodic titanium rods (3-mm diameter) were placed in an electrical cell that filled with 300-mL nitric acid electrolyte (HNO_3_), as shown in Figure 1A. Two cathodes were used to generate liquid plasma discharges and to avoid unbalanced flow, as well as to ensure sufficient hydrogenation effects at the double side of the Ti foam. The strong plasma on cathode surfaces was generated by supplying square wave impulse voltages between anodes and cathodes. A water-cooling machine was used to avoid electrolyte evaporation. The surface color of Ti foam varied from pale gray to charcoal gray after 1 h of liquid plasma treatment. Afterward, the gray titanium foam was put into a beaker filled with 20-mL deionized water and treated with an ultrasonic cleaner for 15 min to obtain a gray liquid, which was then dried with a microwave to obtain gray nanopowders, as shown in Figure 1B. All samples were obtained with the same treatment time (1 h), output voltage (700 V), and frequency (1 kHz) but with different output power. The radiation spectra of liquid plasma from 350 to 560 W were recorded in Figure S1. The resulting sample name was denoted by the output power of the liquid plasma, for example, AT-350 referred to HAm-TiO_2−x_ prepared by applying 350-W output power, AT-420 prepared with 420 W, AT-490 prepared with 490 W, and AT-560 prepared with 560 W.

### 2.3. Preparation of HAm-TiO_2−x_-Coated Glass

HAm-TiO_2−x_ coated glass was prepared by impregnation method. First, to promote the surface hydrophilicity of the glass substrate, a corona discharger (Zhejiang Ruian Zhilin Corona Equipment Co., Ltd., Ruian, China) was used to make surface superhydrophilicity. Second, the treated glass substrate was put into a well-dispersed HAm-TiO_2−x_ liquid (AT-420, 0.1 mg/mL) for 1 min and was then taken out and dried with a microwave oven to deposit a thin HAm-TiO_2−x_ film on the glass.

### 2.4. Characterization

An X-ray powder diffraction instrument (XRD, Rigaku Smartlab, Rigaku, Tokyo, Japan) was used to analyze the crystallinity of HAm-TiO_2−x_. The high-resolution morphology images were acquired by transmission electron microscopy (TEM, JEOL JEM-2100F, JEOL, Tokyo, Japan) with a 200-kV accelerating voltage. A scanning electron microscope (SEM, ZEISS MERLIN, ZEISS, Jena, Germany) was used to characterize the morphology and energy dispersive spectroscopy mapping using 5-kV acceleration voltage. Shimadzu UV-2700 (Shimadzu, Kyoto, Japan) was carried out to measure UV-Vis-diffused reflectance spectroscopy (DRS) at room temperature. A Micromeritics BET analyser (Micromeritics, Norcross, GA, USA) was used to characterize N_2_ Brunauer, Emmett and Teller (BET) surface area and pore volume of HAm-TiO_2−x_. X-ray photoelectron spectrum (XPS, Thermos Escalab 250Xi, Thermo Scientific, Waltham, MA, USA) was used to identify the valence state of surface elements. X-band electron paramagnetic resonance (EPR) was implemented to verify the lattice defects. The surface wetting ability of HAm-TiO_2−x_-coated glass was measured using a contact angle meter (KINO SL200KB, KINO Scientific Instrument Inc., Boston, MA, USA). A fluorescence spectrophotometer (F-7100, Hitachi, Tokyo, Japan) was employed to record the photoluminescence spectra (PL) of HAm-TiO_2−x_ with 365-nm excitation wavelength. Electrochemical workstation (CHI660E, CH Instruments, Inc., Austin, TX, USA) was applied for measurement of photocurrent density. A Thermo Scientific DXR Raman microscope equipped with a 532-nm laser excitation was used to record Raman spectra at room temperature. X-band EPR spectra for active species trapping experiments were applied by using a Bruker EMX PLUS (Bruker, Billerica, MA, USA). In-situ diffused Fourier transform infrared spectroscopy (In-situ DRIFTS, PerkinElmer Spotlight 400, Waltham, MA, USA) was applied during formaldehyde photodegradation. The emitted spectra of liquid plasma discharge were recorded by a spectrometer (Ocean Optics Maya 2000 pro, Ocean Insight, Orlando, FL, USA).

The positron annihilation lifetime spectrum owns a ^22^Na positron emission source and its activity is approximately 2 × 10^6^ Bq. The positrons with a kinetic energy of 0–540 keV are produced when the source undergoes β^+^ decay and, almost simultaneously, emits γ photons (1.28 MeV). Therefore, the appearance of this gamma photon can be regarded as the starting time for generating positrons; the appearance of 0.511 MeV annihilation gamma photons is the end of the positron annihilation event; this interval is the positron lifetime. The radioactive source is sandwiched between the sample to form a sandwich structure with a total of 2 million counts and, herein, the positron annihilation lifetime spectrum is already recorded. The time resolution of the system is approximately 190 ps, with a track width of 12.5 ps.

### 2.5. Photodegradation Experiments of HA-TiO_2−x_

#### 2.5.1. The Dyes Photodegradation of HAm-TiO_2−x_ Nanoparticles and Their Coatings

The photoactivity of HAm-TiO_2−x_ was assessed by photodegradation of rhodamine B (RhB) under visible light (420−800 nm). First, HAm-TiO_2−x_ nano-powders (50 mg) and RhB solution (20 mg/L, 50 mL) were put into a beaker with 60-min magnetic stirring for adsorption–desorption equilibrium. During photodegradation, 1-mL of solution was taken out from the beaker to test its absorbance at 554-nm wavelength via spectrophotometer. To evaluate the stability of HAm-TiO_2−x_, AT-420 was preserved in water for 7 days for the subsequent use under the same experimental conditions. Furthermore, HAm-TiO_2−x_ coating was applied to decompose RhB and methyl blue (MB). For the RhB photodegradation experiment, a 2-mL RhB solution (20 mg/L) was dropped on the HAm-TiO_2−x_ coating (10 × 5 × 0.3 cm^3^), which was then dried in an oven for 10 min; afterward, the treated HAm-TiO_2−x_ coating was irradiated with visible light for 1 h. During the decomposition of MB, HAm-TiO_2−x_ coating (2 × 2 × 0.3 cm^3^) was immersed in a 10-mL MB solution (20 mg/L) to test its visible-light photoactivity.

The in-situ trap of spin-reactive species under visible-light illumination was recorded by the EPR signals of ·O_2_^−^ and ·OH using 5,5-dimethyl-1-pyrroline-N-oxide (DMPO). In addition, in reactive species trapping experiments, the concentration of all sacrifice agents was 0.2 mM/mL, including ammonium oxalate (AO), Fe (II)-EDTA, potassium iodide (KI), p-benzoquinone (BQ), and isopropanol (IPA).

#### 2.5.2. Formaldehyde (HCHO) Photodecomposition Test over HAm-TiO_2−x_

Photocatalytic elimination of HCHO was performed in a sealed box (1 × 1 × 1 m^3^) at room temperature (25 °C, relative humidity = 52%) under visible-light irradiation. The xenon lamp was placed inside the box as the visible-light source, which was equipped with a bandpass filter glass centered at 600 nm (full width at half maximum (FWHM) = 15 nm). The HAm-TiO_2−x_ coating was deposited on a stainless-steel plate (3036 cm^2^) with a loading of 1 mg/cm^2^; afterward, this stainless-steel plate was placed below the xenon lamp. The power density of irradiated light on the stainless-steel plate was 0.136 mW/cm^2^. A certain amount of 38% HCHO was injected into the sealed box; a 7-W fan was used to ensure proper HCHO diffusion. After dark adsorption–desorption equilibrium, the preliminary concentration of HCHO was approximately 0.6 ppm. During photodegradation, an HCHO tester (PPM-HTV, UK) was used to record the real-time HCHO concentrations. The HCHO photocatalytic formaldehyde mineralization efficiency (η) was measured by the equation: η (%) = (C_0_ − C_t_)/C_0_ × 100%, where C_0_ and C_t_ describe instant HCHO concentrations at 0 and t h, respectively. In addition, 365-nm UV light-mediated HCHO photodegradation was performed under the same conditions except for the light source.

Apparent quantum efficiency (AQY) of HCHO photodegradation was defined as Equations (1)–(3) [30]:

(1) $$\mathrm{AQY}=\frac{\mathrm{number}\;\mathrm{of}\;\mathrm{degraded}\;\mathrm{pollutant}\;\mathrm{molecules}}{\mathrm{number}\;\mathrm{of}\;\mathrm{photons}\;\mathrm{entering}\;\mathrm{the}\;\mathrm{reactor}}\;\times \;100\%$$

N_M_ = N_A_ × n_HCHO_ = N_A_ × ((C_0_ − C_t_) × V)/(30 × 1000) (2)

number of photons entering the reactor = I × S/(hc/λ) × t(3)

where N_M_, N_A_, n_HCHO_, C_0_, C_t_, and V represent the number of degraded pollutant molecules, Avogadro’s constant, moles of degraded HCHO, initial HCHO concentration, HCHO concentration of t h, and volume of the box, respectively. While I, S, h, c, λ, and t are illumination intensity, area, Planck’s constant, the vacuum speed of light, the incident light wavelength, and illumination time, respectively.

### 2.6. Theoretical Calculation Methods

To complete all spin-polarization density functional theory (DFT) calculations, with the Perdew–Burke–Ernzerhof (PBE) formulation, the first-principles were employed within the generalized gradient approximation (GGA) [31,32,33]. The projected augmented wave (PAW) potentials were selected to illustrate the ionic cores and took valence electrons into account [34,35]. Through the Gaussian smearing method, partial occupancies of the Kohn–Sham orbitals were used with 0.05-eV width. When the energy change was lower than 10^−4^ eV, the electronic energy was regarded as self-consistent. When the energy change was lower than 0.05 eV Å^−1^, a geometry optimization was identified as convergent; the vacuum spacing from the structural plane was 18 Å. The TiO_2_ (101) amorphous structure was obtained by ab initio first-principles molecular dynamics (AIMD) simulation calculation with 20 ps using the 300 K. In addition, the TiO_2_ (101) crystal structure had been optimized using the AIMD simulations with the 1-fs step and 300 K; this crystal structure was then changed into amorphous TiO_2_ (101). Finally, it was relaxed using DFT calculations in order to obtain the TiO_2_ (101) stable amorphous structure.

## 3. Results and Discussion

### 3.1. Characterization of HAm-TiO_2−x_

**Figure 2:**
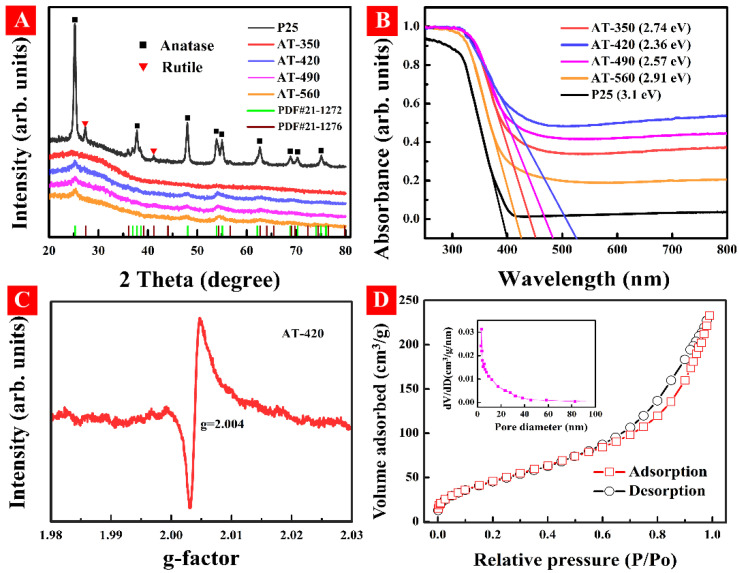
(**A**) The X-ray diffraction (XRD) patterns and (**B**) UV-vis diffuse-reflectance spectra for all as-prepared samples. (**C**) EPR spectrum and (**D**) nitrogen adsorption-desorption isotherms of AT-420 sample, inset is the pore diameter distribution calculated by the BJH method.

Figure 2A exhibits the XRD patterns for all HAm-TiO_2−x_. Here, we provided two XRD cards of crystalline TiO_2_-involved anatase (PDF#21-1272) and rutile (PDF#21-1276). No apparent peaks indexed to crystalline phase are detected, but the weak and broad peaks at (101) facet of all samples compared with that of P25 TiO_2_ indicates that a long-term disordered defect was generated. Figure S2 displays the surface atomic bond environment of all samples by Raman spectra. The observed broad and blue shift of E_g_ mode in our case could be ascribed to the surface oxygen vacancies in HAm-TiO_2−x_ [36]. Figure 2B shows the light response performance of all HAm-TiO_2−x_ using DRS spectra. All samples display enhanced visible light absorption; the bandgaps of AT-350, AT-420, AT-490, and AT-560 are estimated to be 2.74, 2.36, 2.57, and 2.91 eV, respectively. The plots of (αhν)^1/2^ versus hν by using the Kubelka–Munk function were seen in Figure S3. Given the above results, some self-doped defects should exist in HAm-TiO_2−x_. To verify the existence of the surface defect on HAm-TiO_2−x_, EPR spectrum was implemented, as seen in Figure 2C. A sharp EPR signal of g = 2.004 is evidence that the AT-420 sample possessed oxygen vacancies [37]. In addition, Figure 2D illustrates the size and structure of the nanopore using nitrogen adsorption–desorption isotherms. The pore structure in HAm-TiO_2−x_ was irregular, which was proven by the as-obtained typical IV curves with hysteresis loops. It was indicated that micropores and/or mesoporous were formed, owing to the hysteresis with the P/P_0_ range of 0.7–0.9; the pore size was nearly 3.4 nm, as displayed in the inset. The large BET (176.7 cm^2^ g^−1^) and pore volume (0.33 cm^3^ g^−1^) in Ham-TiO_2−x_ both contribute to the large capturing of polluted molecules.

**Figure 3:**
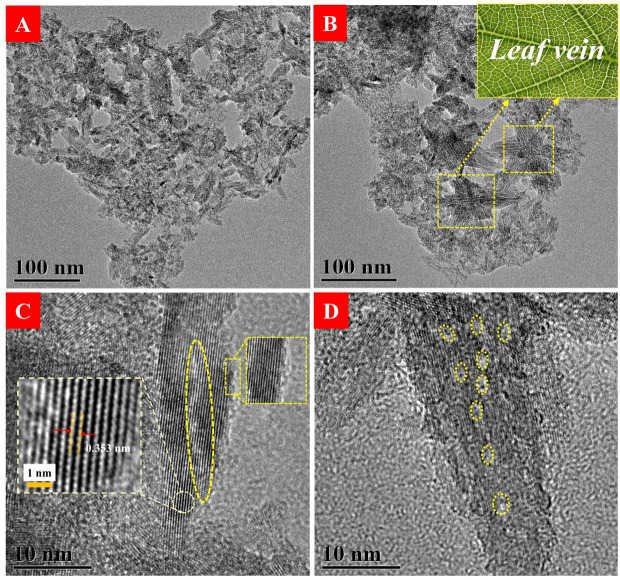
(**A**) Low magnification TEM images of HAm-TiO_2−x_. (**B**) The leaf-vein-shaped nanoparticles in HAm-TiO_2−x_, the inset was a photograph of the leaf vein. (**C**) The curve-like bulk defect marked with yellow oval and the disordered surface marked with yellow rectangle; the crystal plane spacing (d-spacing) was calculated to be 0.353 nm as shown in the left dotted box. (**D**) Some mesoporous structures marked with yellow circles in HAm-TiO_2−x_.

To investigate the morphology characterizations in Ham-TiO_2−x_, TEM was conducted, as displayed in Figure 3. As shown in Figure 3A, HAm-TiO_2−x_ nanoparticles display irregular strip structure. As shown in Figure 3B, lots of textures marked with yellow rectangles exist in the nanoparticles, which seem similar to the leaf vein; these vein-like morphology features are deemed to be the irregular mesoporous structure. As shown in Figure 3C, a curve-liked bulk defect marked with yellow oval was as roughly 20 nm long; furthermore, the disordered surface marked with yellow rectangle proves that surface amorphization with 1–2 nm thickness is prepared after liquid plasma treatment. No long-term range crystalline structure was observed, but some crystal lattices (d = 0.353 nm) of anatase (101) facet existed in HAm-TiO_2−x_, which was in good accordance with the XRD results in Figure 2A. Figure 3D exhibits several nanopores marked with yellow circles with lengths of 2–3 nm, which is classified into mesopore and in good consistency with Barrett-Joyner-Halenda (BJH) result. Overall, HAm-TiO_2−x_ not only possesses the typical characteristics of amorphous materials, including long-term disorder lattice and large specific surface area, but also has broad visible-light harvest and disordered surface on the amorphous core.

### 3.2. Regulation of Surface O_V_ and Bandgap Engineering in HAm-TiO_2−x_

Positron annihilation spectrometry (PAS) is a powerful tool that can be used to characterize the size, type, concentration, and distribution of surface defects in nanomaterials. As listed in Table 1, the relative intensities of three positron lifetime components denoted as τ_1_, τ_2_, and τ_3_ are I_1_, I_2_, and I_3_, respectively. The largest component (τ_3_) is generally regarded as the signal of large voids [38]. The second-largest lifetime component (τ_2_) can be attributed to larger size defects, for instance, O_V_ clusters or surface defects [39]. The smallest component (τ_1_) is responsible for free positrons annihilation with the lattice and at small O_V_ sites on the subsurface [40]. When the output power increased (AT-350 to AT-420), I_1_ decreased, while I_2_ increased, and, notably, I_3_ of AT-420 was too small to detect. This observation indicates that the lattice O_V_ moved to the surface to form surface O_V_, which was then accumulated on the catalyst surface and, thus, shielded the LV signal. With a further increase in output power (from AT-420 to AT-560), I_1_ increased but I_2_ decreased; both the intensities of I_3_ for AT-420 and AT-490 were not detected, whereas I_3_ of AT-560 emerged. It was, therefore, concluded that surface O_V_ moved back to the inner lattice to form subsurface O_V_ in sample AT-560. In conclusion, these results confirm that the output-power-regulated O_V_ not only distributed on the surface but also on the subsurface, rather than merely accumulating on the catalyst surface. More importantly, O_V_ in HAm-TiO_2−x_ existed preferentially at the subsurface if it underwent strong liquid plasma treatment. Compared with the surface O_V_, subsurface and inner-bulk O_V_ are considered quite stable, as the outermost amorphous layer can prevent gradual oxidation by air and water [19,41]. The occurrence of the “roundtrip” path of O_V_ in HAm-TiO_2−x_, as will be discussed further in what follows, could be a result of the synergistic effect of liquid plasma-induced hydrogenation and anodization.

**Figure 4:**
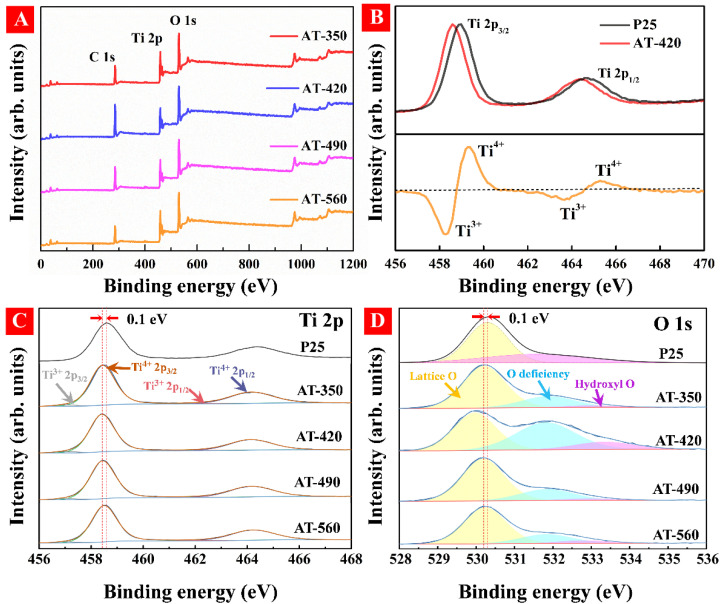
(**A**) Full XPS spectra for all samples, (**B**) Ti 2p spectra of AT-420 and P25 TiO_2_. The deconvoluted Ti 2p (**C**) and O 1s (**D**) spectra for all samples.

**Figure 5:**
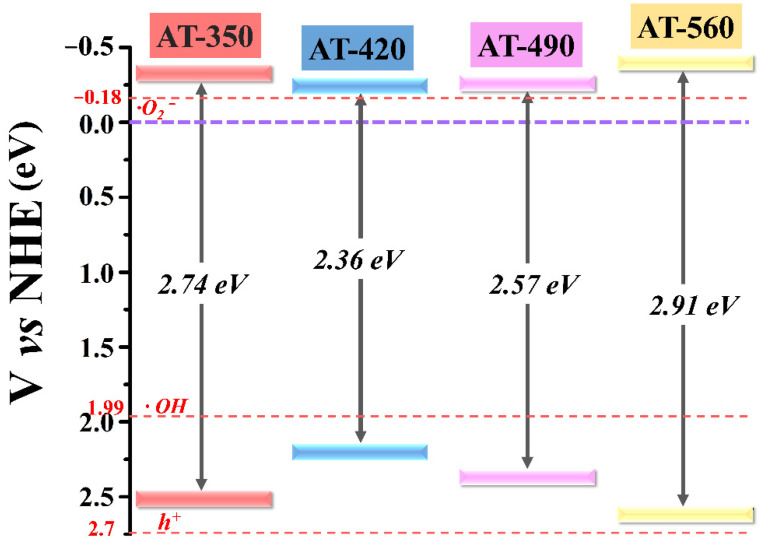
The bandgap structures of all samples.

To inspect the surface chemical states, XPS spectra were performed in HAm-TiO_2−x_. As seen in full XPS spectra of Figure 4A, the lack of obvious differences between these samples demonstrated that the element components were identical. As shown in Figure 4B, P25 TiO_2_ had two typical binding energies of Ti 2p_3/2_ (459.2 eV) and Ti 2p_1/2_ (465.2 eV) attributed to Ti^4+^ peaks [42,43]. In comparison, an apparent shift toward lower binding energies was observed for the sample AT-420, which suggested that Ti^3+^ existed on the disordered surface. We, thus, subtracted the Ti 2p spectra of P25 TiO_2_ from that of sample AT-420, as seen in the bottom of Figure 4B; the resultant orange spectrum curve received two evident peaks (458.2 and 463.7 eV) ascribed to the Ti^3+^ (Ti 2p_3/2_ and Ti 2p_1/2_) [44,45]. To calculate the ratio of Ti^3+^/Ti^4+^, the deconvoluted spectra of Ti 2p are provided in Figure 4C; the ratio was calculated by the areas of Ti^3+^ and Ti^4+^ at Ti 2p_3/2_ [46]. The highest Ti^3+^/Ti^4+^ ratio in AT-420 confirmed the highest concentration of Ti^3+^ in these samples, which is consistent with the PAS results; the detailed data regarding the Ti^3+^/Ti^4+^ ratio are displayed in Table 2. As shown in Figure 4D, three kinds of high-resolution O 1s peaks were observed, which were considered as the lattice oxygen (lattice O, 530.1 eV), surface oxygen defects (O deficiency, 531.9 eV), and hydroxyl group oxygen (hydroxyl O, 533.1 eV) [47,48]. As comparison, a much stronger peak intensity of O deficiency in AT-420 demonstrated a higher content of surface O_V_. Furthermore, the clear shift toward lower binding energy for the lattice O peak observed in sample AT-420 was due to the high-density surface oxygen vacancies [49]. Therefore, as shown in Figure 4B–D, compared with P25 TiO_2_, an apparent blue shift (0.1 eV) in Ti 2p and O 1s spectra were observed, which confirmed the existence of surface defects of Ti^3+^ and O_V_ on the disordered surface. To clarify the effect of Ti^3+^ concentration on bandgap structure modification, the XPS valence band maximum (VBM) spectra of HAm-TiO_2−x_ were investigated, as shown in Figure S4. The estimated VBM value for all samples was recorded in Table 2. Herein, according to Table 2, the band structures for all samples are displayed in Figure 5. It is clear that sample AT-420 exhibited the highest position of the valence band, which could induce a strong redox reaction and higher photoactivity. Notably, the electric potential of h^+^ was higher than all samples, indicating that it is possible to disable UV-light-induced photoactivity in HAm-TiO_2−x_. Here, the output-power-controlled bandgap engineering in HAm-TiO_2−x_ was realized, which was obtained via the regulation of the distribution and the concentration of O_V_.

### 3.3. Effect of Distribution and Concentration of O_V_ on Electrical Structure

**Figure 6:**
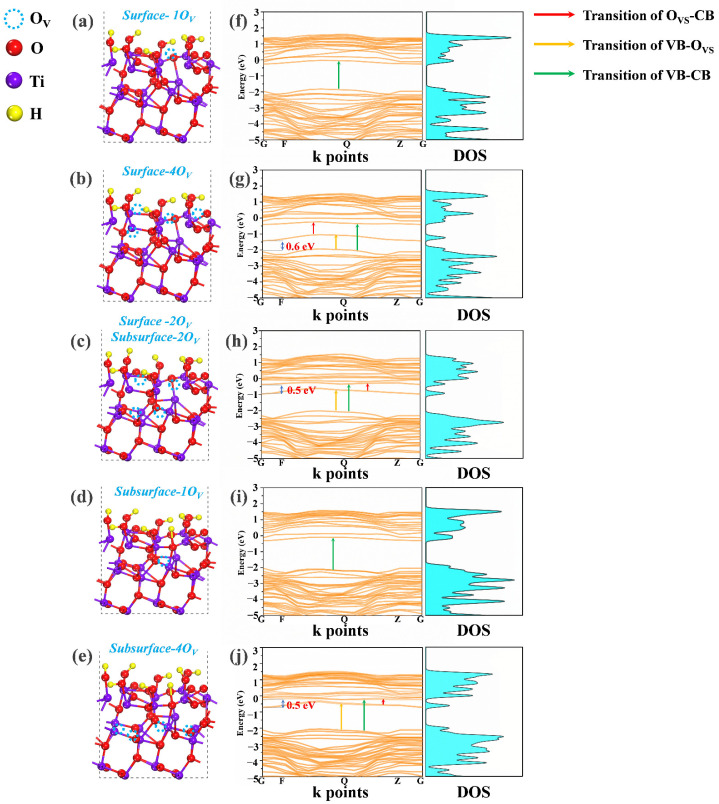
Five kinds of atomic models are shown in (**a**–**e**), respectively, and the corresponding band structure and DOS are shown in (**f**–**j**).

The above PAS and XPS results indicated that the bandgap engineering of HAm-TiO_2−x_ could be directly achieved via the regulation of the distribution and concentration of O_V_. However, an in-depth understanding of the effect of distribution and concentration of O_V_ on the regulation mechanism of the refined bandgap structure was still missing. Thus, DFT calculations regarding the effect of distribution and concentration of O_V_ on the electrical structures of HAm-TiO_2−x_ were carried out here. To balance the distribution and concentration of O_V_, we used five kinds of atomic models, denoted as: Surface-1O_V_, Surface-4O_V_, Surface-2O_V_ and Subsurface-2O_V_, Subsurface-1O_V_, and Subsurface-4O_V_, as depicted in Figure 6a–e, respectively. The naming system used here is such that the model of Surface-1O_V_ represented the case in which one oxygen vacancy is located on the surface, and the model of Surface-2O_V_ and Subsurface-2O_V_ denoted two oxygen vacancies on the surface and two oxygen vacancies at the subsurface. The electronic band structures and density of states (DOS) of all models are displayed in Figure 6f–j. With the increase in the amount of surface O_V_ (Surface-1O_V_ to Surface-4O_V_), deep trap states were generated above the valence band (~0.6 eV), and the flat deep states indicated large effective mass and restricted charge mobility, which led to photocarrier recombination. Owing to the introduction of deep states, some potential transitions may occur, including valence band to O_V_-induced deep states (VB–O_VS_), valence band to conduction band (VB–CB), and O_V_-induced deep states to the conduction band (O_VS_–CB), as depicted in Figure 6g. When the surface O_V_ moved to the inner domain to form subsurface O_V_ (Surface-4O_V_ to Surface-2O_V_ and Subsurface-2O_V_), deep trap states upshifted near the conduction band (~0.5 eV). In comparison with O_VS_-CB transition in the Surface-4O_V_ model, the transition observed in Surface-2O_V_ and Subsurface-2O_V_ was of much lower energy, which could induce a longer wavelength photoresponse, as shown in Figure 6h. When surface O_V_ moved entirely into the inner region (Subsurface-1O_V_), as depicted in Figure 6d, deep trap states disappeared, but several conduction band tails emerged, as shown in Figure 6i, which resulted in a much narrower bandgap. By further increasing subsurface O_V_ (Subsurface-4O_V_), as seen in Figure 6j, deep trap states close to the conduction band (~0.5 eV) reemerged. In summary, the O_V_ concentration determined the existence of deep trap states in the bandgap, and the O_V_ distribution determined their exact location. High concentration of surface O_V_ could introduce the deep trap states, while O_V_ at the subsurface could upshift the deep trap states. On the basis of the PAS results, subsurface O_V_ was a substantial support in HAm-TiO_2−x_, which suggests that the refined electrical structure of HAm-TiO_2−x_ could follow the model of Subsurface-4O_V_, generating deep trap states near the conduction band.

### 3.4. The Photo-Generated Carrier Activity of HAm-TiO_2−x_

**Figure 7:**
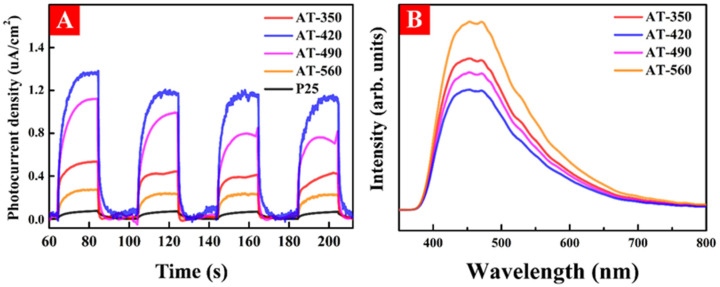
(**A**) The transient photocurrent density and (**B**) photoluminescence spectra for all samples.

To explore the influence of subsurface O_V_ on the production and transferability of photogenerated charge carriers, the transient photocurrent density and PL spectra were explored using visible-light irradiation. As shown in Figure 7A, AT-420 displayed the highest photocurrent density among these samples, revealing the largest photoinduced carrier production and more efficient charge carrier separation. As displayed in Figure 7B, the PL spectra were measured to evaluate the photo-generated e–h pairs separation and transfer efficiency. It is clear that sample AT-420 exhibited the lowest intensity of all the samples, suggesting accelerated photoinduced carrier separation and transfer. Therefore, subsurface O_V_ in HAm-TiO_2−x_ contributed to the generation and transferring ability of photocarriers, which could greatly increase its visible-light photoactivity.

### 3.5. The Visible-Light-Driven Photodegradation of HAm-TiO_2−x_ and Its Coatings

**Figure 8:**
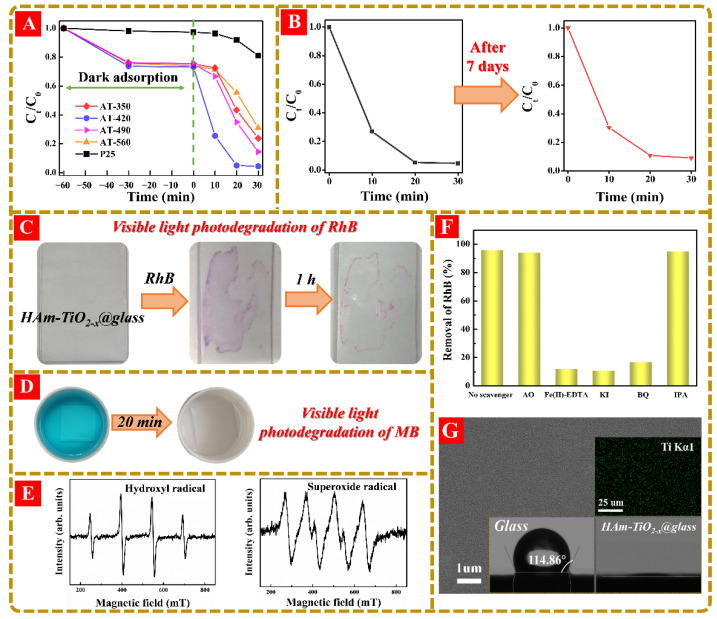
(**A**) The visible-light photodegradation of RhB for all samples and (**B**) the visible-light photodegradation stability test for AT-420. The visible-light photodegradations of (**C**) RhB and (**D**) MB using HAm-TiO_2−x_ coated glass. (**E**) The EPR detection of ·O_2_^−^ and ·OH over AT-420 under visible-light illumination. (**F**) The removal rates of AT-420 in the presence of different scavenging species. (**G**) Large-view SEM picture of HAm-TiO_2−x_ coated glass, the EDS mapping of Ti element and the hydrophilicity test in HAm-TiO_2−x_ coated glass were shown in the inset.

The visible-light photoactivity of HAm-TiO_2−x_ was evaluated by RhB photodegradation, shown in Figure 8A. During the dark adsorption, nearly 27.3% of RhB was removed by adsorption. AT-420 displayed the best performance among these samples, such that RhB photodegradation was accomplished within 20 min, owing to the fact that surface/subsurface O_V_ is generally considered unstable in air and water by the ambient oxidation, leading to faded photoactivity. Herein, to check the stability, AT-420 was put into deionized water for 7 days under ambient conditions for the aging test. Obviously, there was no clear difference between each round when using the aged AT-420 shown in Figure 8B, which proves that this surface/subsurface O_V_ in HAm-TiO_2−x_ was rather stable. In order to extend applications under various complicated environments, photocatalyst nanoparticles were usually deposited on diverse substrates, including glass, plastic film, and fiber. Herein, we urged the use of photocatalytic coating that has highly visible-light driven self-cleaning performance. We deposited HAm-TiO_2−x_ nanoparticles on glass (HAm-TiO_2−x_ @ glass, 10 × 5 × 0.3 cm^3^), which displayed an excellent photocatalytic self-cleaning performance when decomposing the dyes. As shown in Figure 8C, apart from the remaining high concentration “coffee ring” of RhB, most of the area became clean again. In addition, the visible-light photodegradation of methylene blue (MB) was also performed using the coated glass (2 × 2 × 0.3 cm^3^), as shown in Figure 8D, displaying a complete degradation of MB pollution. Therefore, according to the above polluted water photodegradation experiments, both HAm-TiO_2−x_ nanoparticles and its coatings enjoyed high visible photoactivity. Moreover, to detect ·OH and ·O_2_^−^, EPR spin trapping experiments were employed over AT-420, with visible-light irradiation. As observed in Figure 8E, obvious signals of DMPO-OH and DMPO-O_2_^−^ confirmed that ·OH and ·O_2_^−^ were the dominant active oxygen species during photodegradation. Notably, the quantity of ·O_2_^−^ was as much as seven times larger than that of ·OH listed in Table S1, which was generated by the surface O_V_-absorbed electrons and which, simultaneously, verified the large quantities of surface/subsurface O_V_. Actually, except for ·OH and ·O_2_^−^, various reactive species, including ·OH_ads_ (·OH absorbed on the catalyst surface), photoinduced holes (h^+^), electrons, as well as H_2_O_2,_ also participated during the photodegradation process. To explore the contribution from these reactive species, the trapping experiments were carried out over AT-420 using visible-light irradiation. Five kinds of scavengers, including AO, Fe(II)-EDTA, KI, BQ, and IPA were used to probe h^+^, H_2_O_2_, OH_ads_ and electron, ·O_2_^−^, and ·OH in the bulk solution, respectively [50]. As shown in Figure 8F, the visible-light photodegradation rate without scavenger was 95.9%, while, in the presence of AO, Fe(II)-EDTA, KI, BQ, and IPA were 94.1%, 12.2%, 11.3%, 17.8, and 95.3%, respectively. It was, thus, confirmed that ·OH_ads_, ·O_2_^−^, and H_2_O_2_ were the dominated reactive species that contributed to high visible-light photoactivity, which is in good accordance with the EPR spin trapping results. However, h^+^ and ·OH in the bulk solution had almost no influence on photoactivity, which demonstrated that the generation of h^+^ was invalidated by preventing the transition of electrons from the valence band to the conduction band or the O_V_ defect band, and was consistent with the band structures results, shown in Figure 5. The recycling experiment using AT-420 under visible light was carried out; the structural changes of the catalyst after usage are seen in Figure S5. Finally, the surface appearance of HAm-TiO_2−x_ @ glass was characterized by SEM, as seen in Figure 8G. According to 1-μm magnification SEM image, HAm-TiO_2−x_ nanoparticles were uniformly coated on the glass surface, and the EDS mapping with 25-μm magnification also proved that Ti element was uniformly distributed on the glass surface. Increasing surface uniformity of HAm-TiO_2−x_ nanoparticles can contribute to its photocatalytic activity. Due to the distribution of HAm-TiO_2−x_ nanoparticles, the HAm-TiO_2−x_ on the glass showed superhydrophilicity, which could increase the contact area between the catalyst and pollutant molecules, and which, thus, improved the photodegradation performance.

### 3.6. The HCHO Photodegradation Activity under Ambient Environment

**Figure 9:**
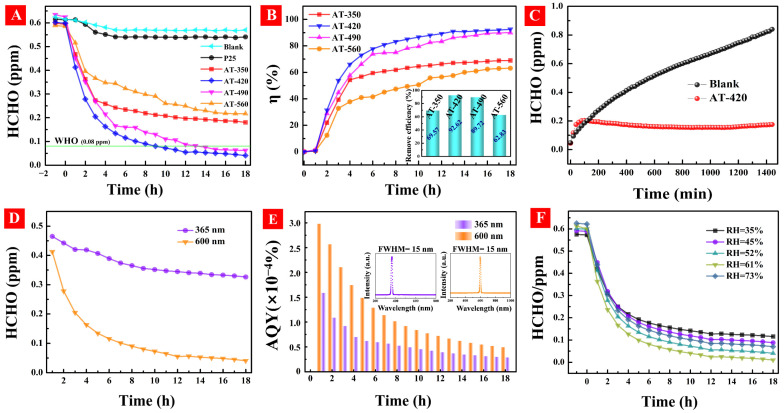
(**A**) Visible-light formaldehyde photodegradation and (**B**) the conversion efficiency with different samples, and the inset was the remove efficiency for all samples. (**C**) Durable test of AT-420 under simulated indoor conditions. (**D**) The HCHO photodegradations under 365-nm and 600-nm light sources using AT-420 sample. (**E**) The AQY of formaldehyde photodegradation with different wavelength light using AT-420 coating, and the inset were the spectra of UV (365 nm, FWHM = 15 nm) and visible (600 nm, FWHM = 15 nm) light. (**F**) The effect of relative humidity for AT-420 coating using 600-nm light source.

To investigate the formaldehyde (HCHO) photodegradation efficiency under ambient environment, a sealed box (1 × 1 × 1 m^3^) was used with simulated indoor conditions inside the box, including low HCHO concentration (~0.6 ppm), air atmosphere, indoor temperature (~25 °C), relative humidity (RH = 45%), and low-power-density irradiation (λ = 600 nm, 0.136 mW/cm^2^). As shown in Figure 9A, HAm-TiO_2−x_ coating was put in the box for 1-h dark adsorption; the HCHO concentrations decreased slightly in the presence of all samples. During the visible light irradiation (0–18 h), gradual HCHO photodegradations were observed and followed a rank: AT-560 < AT-350 < AT-490 < AT-420, which also obeyed the rank of subsurface O_V_ concentration, as shown in PAS results. As an experimental comparison under the same condition, the HCHO concentration only decreased to 0.55 ppm using P25 TiO_2_, owing to its poor visible light photodegradation. Notably, the final HCHO concentrations with AT-420 and AT-490 were both lower than 0.08 ppm, which is the international standard HCHO level for an indoor environment [51]. As shown in Figure 9B, the HCHO conversion efficiency increased rapidly in the initial stage of 0–4 h but reached saturation during 6–18 h; AT-420 exhibited the highest conversion efficiency of 92.62% (inset) among these samples. To verify the stability of HCHO photodegradation, a sustained release formaldehyde source was put in the box. As displayed in Figure 9C, the HCHO concentration linearly increased and, finally, reached 0.8 ppm in the absence of HAm-TiO_2−x_. When AT-420 coating was put in the box, the HCHO concentration, as comparation, sharply increased during the 0–100 min, but trended to a steady value of 0.2 ppm with the increasing time, which suggested that a balance between the HCHO release and the photodegradation was achieved. More importantly, there was no upward tendency of HCHO concentration, demonstrating the strong and stubborn photoactivity of HAm-TiO_2−x_ that can erase the accumulation of HCHO and its by-products on catalyst surface. In addition, UV light of 365 nm assisted HCHO photodegradation, which was carried out using AT-420 coating, as shown in Figure 9D, which unexpectedly displayed poor photoactivity. Furthermore, the AQYs of HCHO photodegradation with different wavelengths of light were measured using AT-420 coating, as shown in Figure 9E. With the 600-nm light source, the AQY in the beginning of 0–2 h was nearly 3 × 10^−4^% but decreased gradually during 3–18 h and reached 0.6 × 10^−4^% eventually. While, with the 365-nm light source, the AQY exhibited a similar change tendency but was much lower than that of the 600-nm light source. Finally, relative humidity, as an important factor that affects HCHO photodegradation, should be explored. As displayed in Figure 9F, when RH during the visible-light photodegradation over AT-420 was 35%, the HCHO elimination efficiency was 80.7%. When increasing RH from 35% to 61%, the elimination efficiency was also enhanced from 80.7% to 98.3%, which could be ascribed to the adsorbed H_2_O on HAm-TiO_2−x_ that contributed to the generation of ·OH radical. When RH arrived at 73%, the HCHO decomposition efficiency, however, decreased to 88.7%, which should be a result of the competitive adsorption between H_2_O and HCHO. Given the above results, HAm-TiO_2−x_ showed high and stable performance in HCHO photocatalytic elimination under the simulated indoor environment.

### 3.7. Investigations on the Surface Reaction Pathway and HCHO Adsorption Mechanism with the Subsurface O_V_

**Figure 10:**
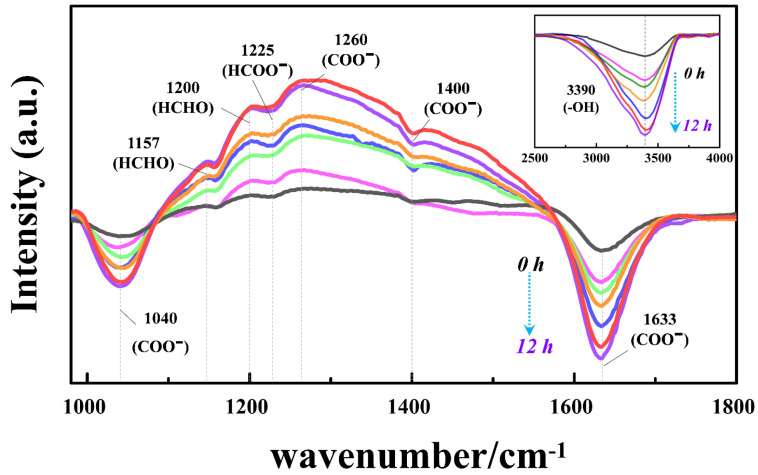
In-situ DRIFTS of AT-420 during the visible-light photodegradation of HCHO.

In-situ DRIFTS analysis was employed to dynamically investigate the reaction pathways of HCHO photodegradation on HAm-TiO_2−x_ surface. As shown in Figure 10, the peaks at 1157 and 1200 cm^−1^ were identified as HCHO [52,53]; the peaks at 1040, 1260, 1400, and 1633 cm^−1^ were ascribed to carbonate species [54,55,56,57]; the peak at 1225 cm^−1^ was considered as a formate species [58], while the peak at 3390 cm^−1^ was considered as surface Ti-OH [23]. Observably, with the increasing irradiation time, the concentrations of all reactants, including formaldehyde, carbonate species, formate species, and surface hydroxyl groups were increased, suggesting that formaldehyde was absorbed on the HAm-TiO_2−x_ surface and oxidized by hydroxyl radicals into formate species and carbonate species. The main intermediates were carbonate species, which suggested the fast oxidation of HCHO and formate species. Thus, we inferred two potential photo-decompose paths of HCHO → HCOOH → H_2_CO_3_ → CO_2_, and HCHO → HCOOH → CO_2_. Unexpectedly, photocatalyst inactivation by this accumulated carbonate species and formate species did not occur, which was proven by the enhancement of surface hydroxyl groups with time. In light of these results and discussion, it was, thus, concluded that HAm-TiO_2−x_ possessed a straightforward reaction pathway and relevant strong photoactivity in HCHO photodegradation.

**Figure 11:**
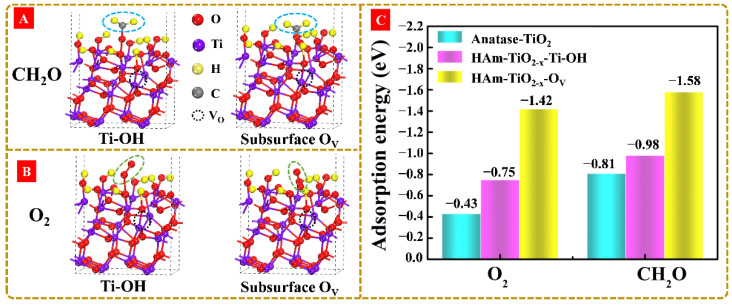
The adsorption models of (**A**) formaldehyde and (**B**) oxygen in the presence of surface hydroxyl groups and subsurface O_V_, respectively. (**C**) The adsorption energies of O_2_ and HCHO over anatase TiO_2_, HAm-TiO_2−x_-OH, and HAm-TiO_2−x_-O_V_.

Generally, the photodegradation of HCHO is assumed to be determined by the synergistic effect of light absorption, photocarriers migration, reactants adsorption, and reaction pathway. Based on the aforementioned characterization results and discussion, HAm-TiO_2−x_ exhibited high performances in visible-light absorption, e–h pairs migration, and straightforward reaction pathways. Herein, reactants adsorption and, especially, the effect of surface hydroxyl group and O_V_ on chemisorb capability of HCHO and O_2_, should be revealed. Here, we constructed two kinds of DFT-simulated adsorption models, including HAm-TiO_2−x_-OH (the effect of surface hydroxyl group) and HAm-TiO_2−x_-O_V_ (the effect of subsurface O_V_) to investigate HCHO adsorption (Figure 11A) and O_2_ adsorption (Figure 11B). Based on the DFT models, the adsorption energies of O_2_ and HCHO on the surfaces of anatase TiO_2_, HAm-TiO_2−x_-OH, and HAm-TiO_2−x_-O_V_ are exhibited in Figure 11C. Anatase TiO_2_ showed the adsorption energies of −0.43 eV (O_2_) and −0.81 eV (HCHO). When using HAm-TiO_2−x_-OH, a lower adsorption energy of O_2_ (−0.75 eV) and HCHO (−0.98 eV) were obtained. As for HAm-TiO_2−x_-O_V_, the corresponding adsorption energies were sharply decreased to −1.42 and −1.58 eV. Therefore, adsorption capabilities of O_2_ and HCHO in HAm-TiO_2−x_ were both largely promoted in the presence of subsurface O_V_, which could significantly promote the capturing of HCHO on the HAm-TiO_2−x_ photocatalyst and improve the HCHO photodegradation efficiency.

### 3.8. The Mechanism of HCHO Photocatalysis and O_V_ Generation over HAm-TiO_2−x_

**Figure 12:**
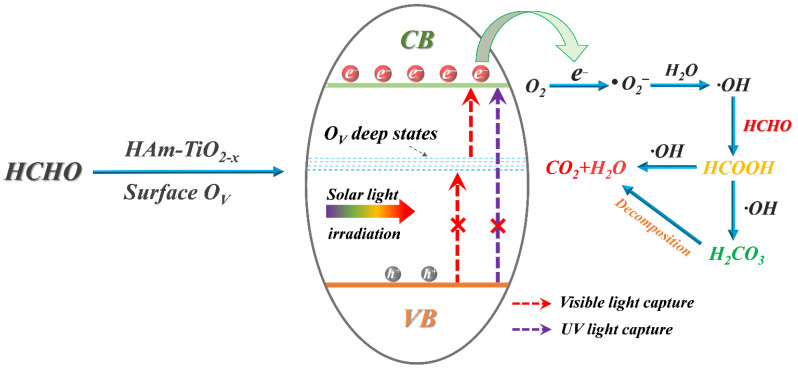
The proposed mechanism for photocatalytic elimination of HCHO with HAm-TiO_2−x_.

Based on the above results and discussions, the photocatalysis mechanism of HCHO purification with HAm-TiO_2−x_ was illustrated in Figure 12. The refined electrical structure of HAm-TiO_2−x_ followed the band structures of the Subsurface-4O_V_ model, as mentioned in Figure 6e, which generated deep trap states close to the conduction band. When solar light irradiation—which has large effective mass—owing to the O_V_-induced midgap trap states, restricts charge mobility, electrons from valence states are hardly transferred from valence band to O_V_-induced deep states or conduction band, leading to bad photoactivity in the UV region. Visible-light capture in HAm-TiO_2−x_ is mainly originated from O_VS_-CB transition, as mentioned in Figure 6. To some extent, HAm-TiO_2−x_, with its self-doped O_V_ defects was, thus, customized for long-wavelength response photocatalysis. On the other hand, with the benefits of abundant surface O_V_, photoinduced electrons had high separation efficiency and long lifetime, resulting in directional migration from the inner lattice to the top surface. Localized surface O_V_ accumulated a considerable number of electrons to react with O_2_ and H_2_O to form·O_2_^−^ and ·OH, which can rapidly decompose the adsorbed HCHO into CO_2_, H_2_O, and intermediates such as HCOOH and H_2_CO_3_. Moreover, contributed by subsurface O_V_ and surface hydroxyl radical (·OH), chemisorb energy of HAm-TiO_2−x_ was much lower than that of crystalline anatase TiO_2_, resulting in the boosted adsorption capability of HCHO.

Finally, the formation mechanism of HAm-TiO_2−x_ nanoparticles should be clarified. In fact, apart from the generation of hydrogen atoms, liquid plasma discharge also produces many kinds of oxidative species including superoxide anion radicals, electrons, hydroxyl radical, hydrogen peroxide, and ultraviolet radiation, which enables strong oxygenation in electrolyte [59,60]. Herein, liquid plasma discharge involved two contrasting effects: liquid plasma hydrogenation and oxidation. Whether hydrogenation or oxidation dominates the whole reaction depends on the output power. Typically, hydrogenation plays a major role at a lower output power. With a proper increase of output power, hydrogenation is simultaneously enhanced and governs the reaction, but, when output power further increases and reaches a threshold, oxidation beyond hydrogenation dominates the whole reaction. In our case, compared with AT-350, a stronger hydrogenation effect was achieved with AT-420 (Figure S1), which resulted in a much higher O_V_ concentration. By further increasing output power (AT-420 to AT-560), surface O_V_ concentration declined, which could be due to the leading role of liquid plasma oxidation over the process. As a result, this alternate effect of hydrogenation and oxidation could also account for the “round-trip” occurrence of O_V_ in HAm-TiO_2−x_, as well as the rank of subsurface O_V_ concentration, listed as AT-560 < AT-350 < AT-490 < AT-420 (as seen in PAS and XPS data).

## 4. Conclusions

In this work, hydrogenated amorphous TiO_2−x_ with high surface area (176.7 cm^2^ g^−1^) was successfully prepared using a liquid plasma-induced hydrogenation strategy. The effect of surface O_V_ engineering on the physico-chemical properties of amorphous TiO_2_ photocatalyst and, in particular, the manipulation of O_V_ concentration and distribution on its electrical structure, was revealed. The optical and electrical properties of HAm-TiO_2−x_ were affected by the surface O_V_ engineering, which could be directly regulated by controlling the output power of liquid plasma. Subsurface O_V_, rather than surface O_V_, induced a disordered surface, which accounted for the narrow bandgap (2.36 eV) by introducing deep trap states under a conduction band (0.5 eV). With the advantages of strong visible-light capture and efficient photocarriers transferring, HAm-TiO_2−x_ and its coatings exhibited strong visible-light photodegradation of polluted water. Furthermore, HAm-TiO_2−x_ was employed to decompose low-concentration formaldehyde under severe indoor environments. Investigations on the surface reaction pathway and formaldehyde adsorption mechanism with the subsurface O_V_ were also carried out. We found that subsurface O_V_ in HAm-TiO_2−x_ could significantly enhance the adsorption energy of HCHO (−1.58 eV) and O_2_ (−1.42 eV), compared with anatase TiO_2_. Based on large amounts of ·OH radicals and strong chemiadsorption capability, HAm-TiO_2−x_ showed high quantum efficiency of 3 × 10^−6^ molecules/photon and photoactivity of 92.6% under visible light (λ = 600 nm). Sustaining growth of surface ·OH radicals guaranteed long-term photocatalytical stability and disabled the surface deactivation of HAm-TiO_2−x_. Owing to its superior photoactivity, extremely low cost, and simple preparation technology, hydrogenated amorphous TiO_2−x_ could be suitable for large production to apply in some practical photocatalytic environment purifications in the future.

## Figures and Tables

**Table 1:** Positron lifetime and relative intensities of HAm-TiO_2−x_, where the O_VL_, O_VS_, and LV are attributed to lattice O_V_, surface O_V_, and large voids, respectively.

Sample	τ_1_ (ns)	O_VL_-I_1_-%	τ_2_ (ns)	O_VS_-I_2_-%	τ_3_ (ns)	LV-I_3_-%
AT-350	0.323	77.1	0.453	22.6	5.142	0.3
AT-420	0.224	10.8	0.363	89.2	/	/
AT-490	0.247	15.4	0.369	84.6	/	/
AT-560	0.329	87.8	0.526	11.8	3.797	0.4

**Table 2:** Bandgap, valence band maximum, and Ti^3+^/Ti^4+^ ratio of the HAm-TiO_2−x_ samples.

Sample	AT-350	AT-420	AT-490	AT-560
Output power (W)	350	420	490	560
Bandgap (eV)	2.74	2.36	2.57	2.91
VBM (eV)	2.48	2.16	2.33	2.58
Ratio of Ti^3+^/Ti^4+^	2.81%	3.01%	2.91%	2.69%

## Data Availability

The data presented in this study are available on request from the corresponding author. The data are not publicly available due to the reason that the data also forms part of an ongoing study.

## Supplementary Materials

The following supporting information can be downloaded at: https://www.mdpi.com/article/10.3390/nano12050742/s1, Figure S1: The emission spectrum of liquid plasma with different output power, (A–D) are obtained by applying the output power of 350, 420, 490, and 560 W, respectively, Figure S2: Raman spectra of all HAm-TiO_2−x_ samples, Figure S3: (A–D) were the Kubelka Munk graphs of AT-350 (2.64 eV), AT-420 (2.24 eV), AT-490 (2.51 eV), and AT-560 (2.89 eV), respectively, Figure S4: X-ray photoelectron spectroscopy valence band spectra of all samples, Figure S5: (A) The recycling experimental of RhB using AT-420 under visible light. (B,C) were EPR and XRD data for AT-420 and catalyst after recycling reaction, respectively. (D) High resolution TEM image of AT-420 after recycling reaction, and the disordered surface was marked with yellow dashed line, Table S1: The amount of **·**O_2_^−^ and **·**OH by employing of 5,5-dimethyl-1-pyrroline-*N*-oxide (DMPO) to in-situ trap the spin-reactive species.

## References

[1] Fujishima A., Rao T.N., Tryk D.A. (2000). Titanium dioxide photocatalysis. J. Photoch. Photobiol. C.

[2] Rao Z., Lu G., Mahmood A., Shi G., Xie X., Sun J. (2021). Deactivation and activation mechanism of TiO_2_ and rGO/Er^3+^-TiO_2_ during flowing gaseous VOCs photodegradation. Appl. Catal. B Environ..

[3] Daghrir R., Drogui P., Robert D. (2013). Modified TiO_2_ for environmental photocatalytic applications: A review. Ind. Eng. Chem. Res..

[4] Luciani G., Imparato C., Vitiello G. (2020). Photosensitive hybrid nanostructured materials: The big challenges for sunlight capture. Catalysts.

[5] Janczarek M., Endo-Kimura M., Raja-Mogan T., Kowalska E., Garg S., Chandra A. (2022). The role of oxygen vacancy and other defects for activity enhancement. Green Photocatalytic Semiconductors: Recent Advances and Applications.

[6] Hao L., Huang H., Zhang Y., Ma T. (2021). Oxygen vacant semiconductor photocatalysts. Adv. Funct. Mater..

[7] Zhang Y., Xu Z., Li G., Huang X., Hao W., Bi Y. (2019). Direct observation of oxygen vacancy self-healing on TiO_2_ photocatalysts for solar water splitting. Angew. Chem. Int. Edit..

[8] Li J., Zhang M., Guan Z., Li Q., He C., Yang J. (2017). Synergistic effect of surface and bulk single-electron-trapped oxygen vacancy of TiO_2_ in the photocatalytic reduction of CO_2_. Appl. Catal. B Environ..

[9] Lin L., Ma Y., Wu J., Pang F., Ge J., Sui S., Yao Y., Qi R., Cheng Y., Duan C. (2019). Origin of photocatalytic activity in Ti^4+^/Ti^3+^ core–shell titanium oxide nanocrystals. J. Phys. Chem. C.

[10] Mao J., An X., Gu Z., Zhou J., Liu H., Qu J. (2020). Visualizing the interfacial charge transfer between photoactive microcystis aeruginosa and hydrogenated TiO_2_. Environ. Sci. Technol..

[11] Li J., Weng B., Cai S., Chen J., Jia H., Xu Y. (2018). Efficient promotion of charge transfer and separation in hydrogenated TiO_2_/WO_3_ with rich surface-oxygen-vacancies for photodecomposition of gaseous toluene. J. Hazard. Mater..

[12] Yan Y., Cheng X., Zhang W., Chen G., Li H., Konkin A., Sun Z., Sun S., Wang D., Schaaf P. (2019). Plasma hydrogenated TiO_2_/Nickel foam as an efficient bifunctional electrocatalyst for overall water splitting. ACS Sustain. Chem. Eng..

[13] Zhang K., Park J.H. (2017). Surface localization of defects in black TiO_2_: Enhancing photoactivity or reactivity. J. Phys. Chem. Lett..

[14] Chen X., Liu L., Huang F. (2015). Black titanium dioxide (TiO_2_) nanomaterials. Chem. Soc. Rev..

[15] Wang G., Wang H., Ling Y., Tang Y., Yang X., Fitzmorris R.C., Wang C., Zhang J.Z., Li Y. (2011). Hydrogen-treated TiO_2_ nanowire arrays for photoelectrochemical water splitting. Nano Lett..

[16] Danyliuk N., Tatarchuk T., Kannan K., Shyichuk A. (2021). Optimization of TiO_2_-P25 photocatalyst dose and H_2_O_2_ concentration for advanced photo-oxidation using smartphone-based colorimetry. Water Sci. Technol..

[17] Tatarchuk T., Danyliuk N., Shyichuk A., Macyk W., Naushad M. (2021). Photocatalytic degradation of dyes using rutile TiO_2_ synthesized by reverse micelle and low temperature methods: Real-time monitoring of the degradation kinetics. J. Mol. Liq..

[18] Kang J., Zhang Y., Chai Z., Qiu X., Cao X., Zhang P., Teobaldi G., Liu L., Guo L. (2021). Amorphous domains in black titanium dioxide. Adv. Mater..

[19] Wang B., Biesold G.M., Zhang M., Lin Z. (2021). Amorphous inorganic semiconductors for the development of solar cell, photoelectrocatalytic and photocatalytic applications. Chem. Soc. Rev..

[20] Sun S., Song P., Cui J., Liang S. (2019). Amorphous TiO_2_ nanostructures: Synthesis, fundamental properties and photocatalytic applications. Catal. Sci. Technol..

[21] Cheng L., Li B., Cheng Q., Baldwin A.N., Shang Y. (2017). Investigations of indoor air quality of large department store buildings in China based on field measurements. Build. Environ..

[22] Chang T., Wang J., Lu J., Shen Z., Huang Y., Sun J., Xu H., Wang X., Ren D., Cao J. (2019). Evaluation of indoor air pollution during decorating process and inhalation health risks in Xi’an, China: A case study. Aerosol Air Qual. Res..

[23] He M., Ji J., Liu B., Huang H. (2019). Reduced TiO_2_ with tunable oxygen vacancies for catalytic oxidation of formaldehyde at room temperature. Appl. Surf. Sci..

[24] Zhu M., Muhammad Y., Hu P., Wang B., Wu Y., Sun X., Tong Z., Zhao Z. (2018). Enhanced interfacial contact of dopamine bridged melamine-graphene/TiO_2_ nano-capsules for efficient photocatalytic degradation of gaseous formaldehyde. Appl. Catal. B Environ..

[25] Huang Q., Hu Y., Pei Y., Zhang J., Fu M. (2019). In situ synthesis of TiO_2_@NH_2_-MIL-125 composites for use in combined adsorption and photocatalytic degradation of formaldehyde. Appl. Catal. B Environ..

[26] Li Y., Chen X., Wang C., Zhang C., He H. (2018). Sodium Enhances Ir/TiO_2_ activity for catalytic oxidation of formaldehyde at ambient temperature. ACS Catal..

[27] Li X., Li H., Huang Y., Cao J., Huang T., Li R., Zhang Q., Lee S.-c., Ho W. (2022). Exploring the photocatalytic conversion mechanism of gaseous formaldehyde degradation on TiO_2–x_-O_V_ surface. J. Hazard. Mater..

[28] He F., Jeon W., Choi W. (2021). Photocatalytic air purification mimicking the self-cleaning process of the atmosphere. Nature Commun..

[29] Wang X., Hong S., Lian H., Zhan X., Cheng M., Huang Z., Manzo M., Cai L., Nadda A., Le Q.V. (2021). Photocatalytic degradation of surface-coated tourmaline-titanium dioxide for self-cleaning of formaldehyde emitted from furniture. J. Hazard. Mater..

[30] Deng X.-Q., Zhu X., Sun Z.-G., Li X.-S., Liu J.-L., Shi C., Zhu A.-M. (2016). Exceptional activity for photocatalytic mineralization of formaldehyde over amorphous titania nanofilms. Chem. Eng. J..

[31] Kresse G., Furthmüller J. (1996). Efficiency of ab-initio total energy calculations for metals and semiconductors using a plane-wave basis set. Comp. Mater. Sci..

[32] Kresse G., Furthmüller J. (1996). Efficient iterative schemes for ab initio total-energy calculations using a plane-wave basis set. Phys. Rev. B.

[33] Perdew J.P., Burke K., Ernzerhof M. (1996). Generalized gradient approximation made simple. Phys. Rev. Lett..

[34] Kresse G., Joubert D. (1999). From ultrasoft pseudopotentials to the projector augmented-wave method. Phys. Rev. B.

[35] Blöchl P.E. (1994). Projector augmented-wave method. Phys. Rev. B.

[36] Choudhury B., Choudhury A. (2013). Oxygen vacancy and dopant concentration dependent magnetic properties of Mn doped TiO_2_ nanoparticle. Curr. Appl. Phys..

[37] Huang H., Hou X., Xiao J., Zhao L., Huang Q., Chen H., Li Y. (2019). Effect of annealing atmosphere on the performance of TiO_2_ nanorod arrays in photoelectrochemical water splitting. Catal. Today.

[38] Dutta S., Chattopadhyay S., Jana D., Banerjee A., Manik S., Pradhan S.K., Sutradhar M., Sarkar A. (2006). Annealing effect on nano-ZnO powder studied from positron lifetime and optical absorption spectroscopy. J. Appl. Phys..

[39] He Y., Dulub O., Cheng H., Selloni A., Diebold U. (2009). Evidence for the predominance of subsurface defects on reduced anatase TiO_2_ (101). Phys. Rev. Lett..

[40] Sun W., Li Y., Shi W., Zhao X., Fang P. (2011). Formation of AgI/TiO_2_ nanocomposite leads to excellent thermochromic reversibility and photostability. J. Mater. Chem..

[41] Liu Y., Chen P., Fan Y., Fan Y., Shi X., Cui G., Tang B. (2020). Grey rutile TiO_2_ with long-term photocatalytic activity synthesized via two-step calcination. Nanomaterials.

[42] Huang J., Yang K., Zhang Z., Yang L., Hirano S. (2017). Layered perovskite LiEuTiO_4_ as a 0.8 V lithium intercalation electrode. Chem. Commun..

[43] Lee J.W., Moon B.M., Lee K.M., Kim Y.H., Park H.G., Lim J.H., Oh B.Y., Kim B.Y., Hwang J.Y., Ok C.H. (2010). Homogeneous liquid crystal orientation on ion beam exposure TiO_2_ surfaces depending on an anisotropic dipole field. Liq. Cryst..

[44] Shi C., Qi H., Sun Z., Qu K., Huang Z., Li J., Dong M., Guo Z. (2020). Carbon dot-sensitized urchin-like Ti^3+^ self-doped TiO_2_ photocatalysts with enhanced photoredox ability for highly efficient removal of Cr^6+^ and RhB. J. Mater. Chem. C.

[45] Li G., Lian Z., Li X., Xu Y., Wang W., Zhang D., Tian F., Li H. (2015). Ionothermal synthesis of black Ti^3+^-doped single-crystal TiO_2_ as an active photocatalyst for pollutant degradation and H_2_ generation. J. Mater. Chem. A.

[46] Guillemot F., Porté M.C., Labrugère C., Baquey C. (2002). Ti^4+^ to Ti^3+^ conversion of TiO_2_ uppermost layer by low-temperature vacuum annealing: Interest for titanium biomedical applications. J. Colloid Interf. Sci..

[47] Hu Z., Yu J.C., Ming T., Wang J. (2015). A wide-spectrum-responsive TiO_2_ photoanode for photoelectrochemical cells. Appl. Catal. B Environ..

[48] Qian A., Hyeon S.E., Seo J.Y., Chung C.-H. (2018). Capacitance changes associated with cation-transport in free-standing flexible Ti_3_C_2_T_x_ (T=O, F, OH) MXene film electrodes. Electrochim. Acta.

[49] Qiu H., Ma X., Sun C., Zhao B., Chen F. (2020). Surface oxygen vacancies enriched Pt/TiO_2_ synthesized with a defect migration strategy for superior photocatalytic activity. Appl. Surf. Sci..

[50] Xu K., Yang X., Sun D., Yang X., Zhou Y., Li W., Yang Q., Yang X., Li R., Feng J. (2019). Enhanced visible-light driven photocatalytic performances over LaFeO_3_/NiO modified porous g-C_3_N_4_ nanosheets. Nano.

[51] Gu Y.H., Fujimiya Y., Kunugita N. (2008). Long-term exposure to gaseous formaldehyde promotes allergen-specific IgE-mediated immune responses in a murine model. Hum. Exp. Toxicol..

[52] Alminshid A.H., Abbas M.N., Alalwan H.A., Sultan A.J., Kadhom M.A. (2021). Aldol condensation reaction of acetone on MgO nanoparticles surface: An in-situ drift investigation. Mol. Catal..

[53] Li J., Cui W., Chen P., Dong X.A., Chu Y., Sheng J., Zhang Y., Wang Z., Dong F. (2020). Unraveling the mechanism of binary channel reactions in photocatalytic formaldehyde decomposition for promoted mineralization. Appl. Catal. B Environ..

[54] Jodłowski P.J., Jędrzejczyk R.J., Chlebda D., Gierada M., Łojewska J. (2017). In situ spectroscopic studies of methane catalytic combustion over Co, Ce, and Pd mixed oxides deposited on a steel surface. J. Catal..

[55] Song S., Lu C., Wu X., Jiang S., Sun C., Le Z. (2018). Strong base g-C_3_N_4_ with perfect structure for photocatalytically eliminating formaldehyde under visible-light irradiation. Appl. Catal. B Environ..

[56] Lu J., Zhong J., Ren Q., Li J., Song L., Mo S., Zhang M., Chen P., Fu M., Ye D. (2021). Construction of Cu-Ce interface for boosting toluene oxidation: Study of Cu-Ce interaction and intermediates identified by in situ DRIFTS. Chin. Chem. Lett..

[57] Xu P., Xu T., Yu H., Zheng D., Li X. MOF (metal-organic framework) nanomaterial for 400 ppb-concentration detectable xylene gas sensors. Proceedings of the 2017 IEEE 30th International Conference on Micro Electro Mechanical Systems (MEMS).

[58] Hu Z., Yang C., Lv K., Li X., Li Q., Fan J. (2020). Single atomic Au induced dramatic promotion of the photocatalytic activity of TiO_2_ hollow microspheres. Chem. Commun..

[59] Jiang B., Zheng J., Qiu S., Wu M., Zhang Q., Yan Z., Xue Q. (2014). Review on electrical discharge plasma technology for wastewater remediation. Chem. Eng. J..

[60] Oinuma G., Nayak G., Du Y., Bruggeman P.J. (2020). Controlled plasma—Droplet interactions: A quantitative study of OH transfer in plasma–liquid interaction. Plasma Sources Sci. Technol..

