# Supplementary material for: Hydrogenated Amorphous Titania with Engineered Surface Oxygen Vacancy for Efficient Formaldehyde and Dye Removals under Visible-Light Irradiation
Source: Nanomaterials (Basel). 2022 Feb 22;12(5):742. doi: 10.3390/nano12050742 (PMC8911576; doi:10.3390/nano12050742)
Supplement: Supplementary file 1 [file nanomaterials-12-00742-s001.zip › nanomaterials-1565474-supplementary.pdf]

nanomaterials
MDPI

Supplementary Materials

Hydrogenated Amorphous Titania with Engineered Surface Oxygen Vacancy for Efficient Formaldehyde and Dye Removals under Visible-Light Irradiation

Guang Feng [1,2], Mengyun Hu [1,2,3], Botao Wu [3], Shencheng Shi [3], Shuai Yuan [1,2], Yanan Li [3] and Heping Zeng [2,3,*]

[1] School of Optical-Electrical and Computer Engineering, University of Shanghai for Science and Technology, Shanghai 200093, China; 191380035@st.usst.edu.cn (G.F.); myhu@phy.ecnu.edu.cn (M.H.); syuan@usst.edu.cn (S.Y.)
[2] Chongqing Key Laboratory of Precision Optics, Chongqing Institute of East China Normal University, Chongqing 401120, China
[3] State Key Laboratory of Precision Spectroscopy, East China Normal University, Shanghai 200062, China; btwu@phy.ecnu.edu.cn (B.W.); 51190920037@stu.ecnu.edu.cn (S.S.); 51200920008@stu.ecnu.edu.cn (Y.L.)
* Correspondence: hpzeng@phy.ecnu.edu.cn

S1. Results and Discussion

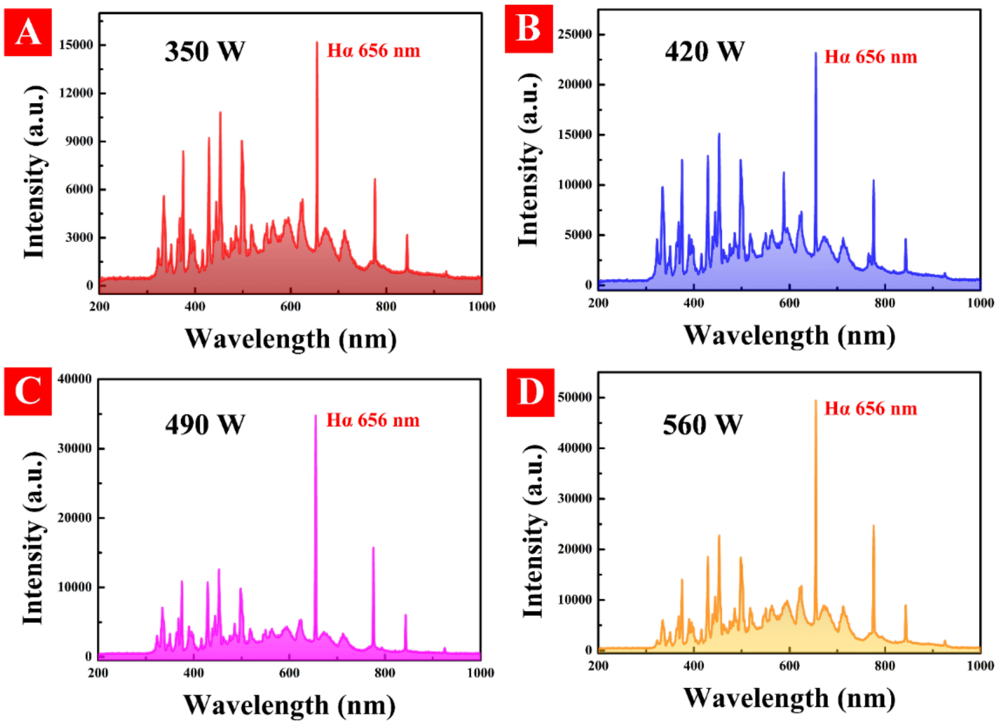

Figure S1. The emission spectrum of liquid plasma with different output power, (**A**), (**B**), (**C**), and (**D**) are obtained by applying the output power of 350, 420, 490, and 560 W, respectively.

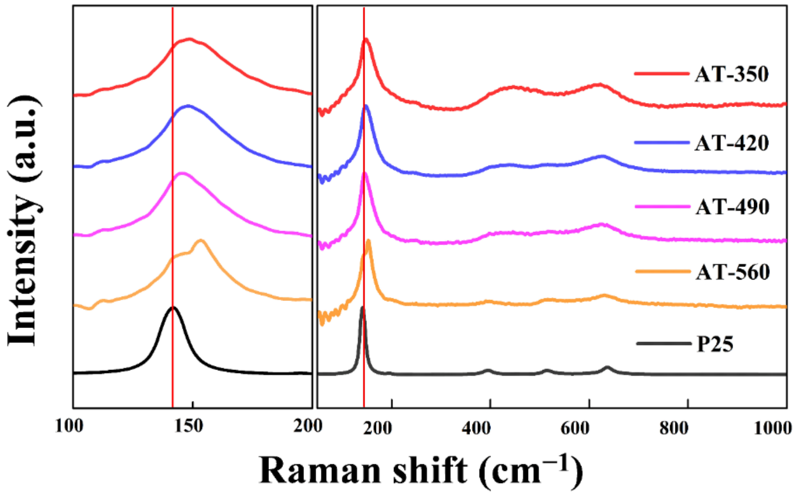

Figure S2. Raman spectra of all HAm-TiO_{2-x} samples.

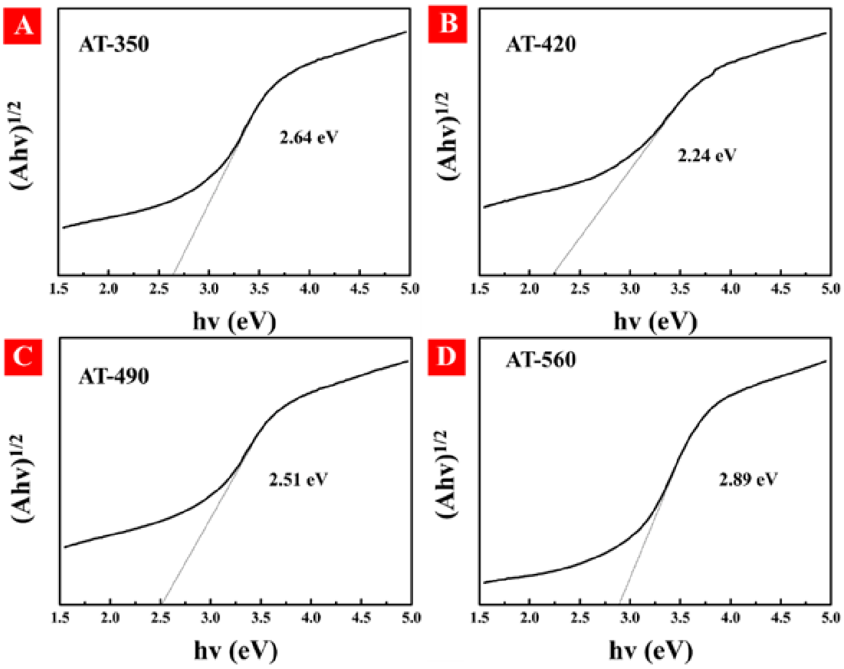

Figure S3. (**A**), (**B**), (**C**) and (**D**) were the Kubelka Munk graphs of AT-350 (2.64 eV), AT-420 (2.24 eV), AT-490 (2.51 eV), and AT-560 (2.89 eV), respectively.

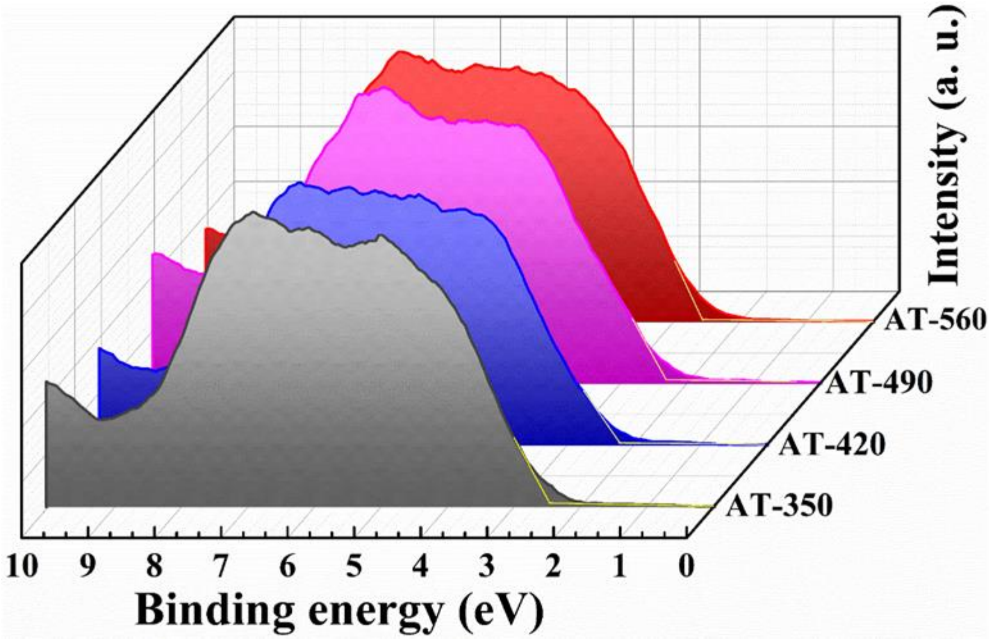

Figure S4. X-ray photoelectron spectroscopy valence band spectra of all samples.

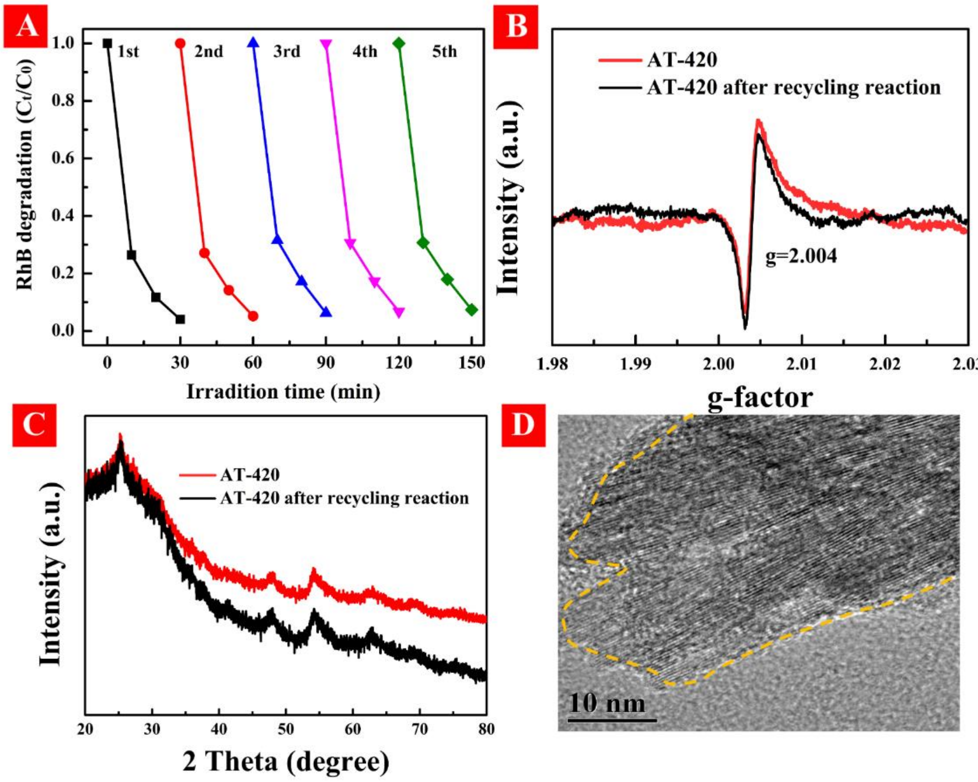

Figure S5. (**A**) The recycling experimental of RhB using AT-420 under visible light. (**B**) and (**C**) were EPR and XRD data for AT-420 and catalyst after recycling reaction, respectively. (**D**) High resolution TEM image of AT-420 after recycling reaction, and the disordered surface was marked with yellow dashed line.

As shown in Figure S5A, no obvious decline was observed after 5-times recycling reaction. Meanwhile, the XRD, TEM and EPR data after recycling reaction were also provided. As shown in Figure S5B, the surface O_V (g = 2.004) is still observed in HAm-TiO_{2-x}, and the crystal structure remains unchanged and is still amorphous phase shown in Figure S5C. The high resolution TEM of AT-420 after recycling reaction was seen in Figure

S5D, the disordered surface marked with yellow dashed line is presented which suggests that the existence of O_V defects, and was also consistent with EPR data in Figure S5B. Therefore, the stability of surface O_V of HAm-TiO_{2-x} accounted for its stable recycling photodegradation.

Table S1. The amount of $\cdot O_2^-$ and $\cdot OH$ by employing of 5,5-dimethyl-1-pyrroline-*N*-oxide (DMPO) to in-situ trap the spin-reactive species.

**Sample**	**Spins / mm^3**	**M**	**Spins**
$\cdot OH$	2.653×10^{12}	4.405×10^{-6}	5.834×10^{13}
$\cdot O_2^-$	1.4×10^{13}	2.341×10^{-5}	3.1×10^{14}

Notes: "Spins / mm^3" is the number of spins per unit volume, "M" is the molarity, and "spins" is the total number of spins, and thus the free radicals can be estimated with spins/mm^3.
